# Incremental prognostic value of intensity-weighted regional calcification scoring using contrast CT imaging in TAVR

**DOI:** 10.1093/ehjimp/qyad027

**Published:** 2023-09-29

**Authors:** Mohamed Abdelkhalek, Nikrouz Bahadormanesh, Javier Ganame, Zahra Keshavarz-Motamed

**Affiliations:** School of Biomedical Engineering, McMaster University, 1280 Main St W, Hamilton, ON L8S4L8, Canada; Department of Mechanical Engineering, McMaster University, 1280 Main St W, Hamilton, ON L8S4L8, Canada; Department of Medicine, McMaster University, Hamilton, 1280 Main St W, Hamilton, ON L8S4L8, Canada; School of Biomedical Engineering, McMaster University, 1280 Main St W, Hamilton, ON L8S4L8, Canada; Department of Mechanical Engineering, McMaster University, 1280 Main St W, Hamilton, ON L8S4L8, Canada; School of Computational Science and Engineering, McMaster University, 1280 Main St W, Hamilton, ON L8S4L8, Canada

**Keywords:** aortic stenosis, aortic valve calcification, computed tomography, contrast-enhanced CT, transcatheter aortic valve replacement

## Abstract

**Aims:**

Aortic valve calcification scoring plays an important role in predicting outcomes of transcatheter aortic valve replacement (TAVR). However, the impact of relative calcific density and its causal effect on peri-procedural complications due to sub-optimal valve expansion remains limited. This study aims to investigate the prognostic power of quantifying regional calcification in the device landing zone in the context of peri-procedural events and post-procedural complications based on pre-operative contrast computed tomography angiography (CCTA) images. Assess the effect of calcification on post-procedural device expansion and final configuration.

**Methods and results:**

We introduce a novel patient invariant topographic scheme for quantifying the location and relative density of landing zone calcification. The calcification was detected on CCTA images based on a recently developed method using automatic minimization of the false positive rate between aortic lumen and calcific segments. Multinomial logistic regression model evaluation and ROC curve analysis showed excellent classification power for predicting paravalvular leakage [area under the curve (AUC) = 0.8; *P* < 0.001] and balloon pre-dilation (AUC = 0.907; *P* < 0.001). The model exhibited an acceptable classification ability for left bundle branch block (AUC = 0.748; *P* < 0.001) and balloon post-dilation (AUC = 0.75; *P* < 0.001). Notably, all evaluated models were significantly superior to alternative models that did not include intensity-weighted regional volume scoring.

**Conclusions:**

TAVR planning based on contrast computed tomography images can benefit from detailed location, quantity, and density contribution of calcific deposits in the device landing zone. Those parameters could be employed to stratify patients who need a more personalized approach during TAVR planning, predict peri-procedural complications, and indicate patients for follow-up monitoring.

## Graphical Abstract

**Graphical abstract:**
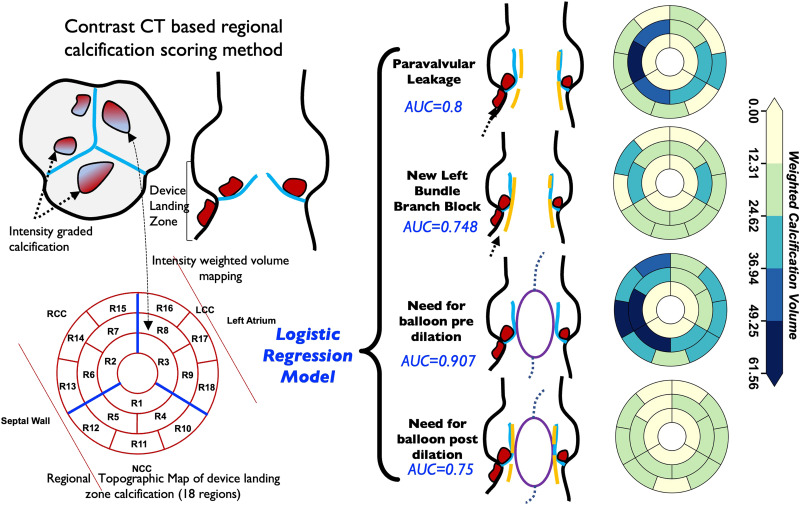

## Introduction

Symptomatic severe calcific aortic stenosis is the most common valvular disease requiring surgery or intervention, and prevalence continues to rise as the population ages.^1–4^ In recent years, transcatheter aortic valve replacement (TAVR), a minimally invasive alternative to surgical aortic valve replacement (SAVR), has become more common and is the most widely used treatment for severe aortic valve (AV) stenosis.^1,5^ Most recently, utilization of TAVR has been expanded to lower-risk candidates.^4^ Unlike SAVR, during a TAVR procedure, the perivalvular area is not directly explored by the operator, and the variability in AV anatomy makes correct sizing, selecting optimal landing zone location and sufficient balloon expansion a more challenging task.^6^ This process is further complicated by the presence of calcifications on the cusps, annulus, and left ventricular outflow tract (LVOT). The combined calcification of these regions commonly referred to as device landing zone calcification^6^ (DLZ) exhibit patient specific variations in relative density and spatial distribution, which might play a key role in prognosing short- and long-term outcomes.^4,6^

Incorrect prosthetic device sizing, over/under balloon expansion, and eccentric expansion in the TAVR landing zone are all associated with worsening outcomes leading to various degrees of paravalvular leakage/regurgitation (PVL/PVR).^7^ In addition, due to the proximity of the device with the membranous interventricular septum, left bundle branch block (LBBB) or complete atrioventricular block can occur post-TAVR.^4,6^ An increased implantation depth, annular/LVOT(ALVOT) calcification, and asymmetric calcific distributions are considered important risk factors for predicting the need for pacemaker implementation post-TAVR.^8,9^

In terms of procedural events, the TAVR procedure has been changed as well, with a focus on less complicated techniques that involve less frequent balloon pre-dilation (preD) of the native calcified leaflets and post-dilation (postD) of TAVR device.^10^ Consequently, the TAVR procedure needs a more individualized approach.^4^ Proper quantification of calcification patterns may be the key to such individualized approaches. Calcium deposits and their density may act as mechanical obstacles that hinder stent expansion affecting the final configuration of the device.^11^

The link between calcification patterns and clinical consideration during TAVR and post-TAVR outcomes is strongly proven in single and multi-centre studies.^4,12^ However, we believe that there is an existing gap in the literature about the detailed assessment of calcium deposits and their impact. Contrast computed tomography angiography (CCTA) is recommended for TAVR planning. It can provide detailed geometric and structural information about landing zone calcium deposition, thanks to its superior spatial and contrast resolution,^13^ However, assessment of calcification using CCTA is challenging due to variability in contrast attenuation,^2^ lack of standardized methodology in determining suitable calcific thresholds,^7^ and poorly defined relationship between contrast HU intensity and tissue density.^2^

In that respect, we developed a novel semi-automatic computational framework based on CCTA images. Based on a previously^14^ developed technique [false positive rate (FPR)] to detect calcification in the presence of contrast, we further enhanced the FPR method to quantify the geometrical and structural characteristics of calcium deposits in pre- and post-TAVR images. The method allows us to determine the relative density in addition to the spatial location of the deposition in the form of patient invariant topographic maps, which was then used to group and analyse patient groups with adverse outcomes or procedural events. To the best of the authors’ knowledge, no study has yet shed light on the causal link between the relative density and 3D spatial distribution of calcium deposits in the context of balloon dilation events and short-term post-TAVR complications.

## Methods

### Study population

This retrospective study reviewed clinical and CT data of patients who underwent TAVR with a balloon-expandable Sapien 3, Sapien 3 Ultra, or a Sapien XT transcatheter heart valve (THV) (Edwards Lifesciences, Irvine, CA). Data were acquired from two medical centres (Hamilton General Hospital, Ontario, Canada, *n* = 111 between 2020 and 2022; St. Paul’s Hospital, Vancouver, Canada, *n* = 22 between 2014 and 2015). Patients included in the analysis were required to have undergone gated contrast CT for TAVR planning and 2D Doppler echocardiography assessment before and after intervention. After a review of 251 patients evaluated for TAVR, 133 patients were included in the study. Patients with bicuspid valve morphology, self-expandable devices, those treated for surgical aortic bioprosthesis degeneration (i.e. valve-in-valve), and those with unsuccessful devices as per VARC-3 (Valve Academic Research Consortium 3) criteria were excluded.^15^ No patients were excluded based on image quality. The procedural access route, THV type, and sizing were determined by the local heart teams on basis of annular area and qualitative calcification grade as per recommended SCCT guidelines.^13^ Among the study cohort, 33 patients underwent CT follow-up 9–12 months after intervention. Follow-up CT was performed on patients where there was concern of adverse events^13^ such as valve thrombosis, infective endocarditis, or structural degeneration based on echocardiographic follow-up. Waiver of informed consent and data transfer was approved by the Institutional Review Boards of the respective institutions (iREB). Data collection and clinical measurements were performed by operators blinded to the objectives and contents of this study. Standard measurements were performed per relevant guidelines and regulations including guidelines of the American College of Cardiology and American Heart Association. Demographic and peri-procedural notes were collected from the patients’ medical records (see *Table 1* for patient characteristics).

**Table 1:** Baseline patient characteristics of study cohort (*n* = 133)

Parameter	Category	Value
Age (years)—median [interquartile range]		82 [76–86]
Sex—*n* (%)	Female	56 (42.1%)
	Male	77 (57.9%)
THV size—*n* (%)	20 mm	5 (3.8%)
	23 mm	31 (23.3%)
	26 mm	63 (47.4%)
	29 mm	34 (25.6%)
THV type—*n* (%)*_n_*_= 134_	Sapien 3	67 (50.4%)
	Sapien Ultra	44 (33.1%)
	Sapien XT	22 (16.5%)
NYHA class—*n* (%)	I	4 (3%)
	II	56 (42.1%)
	III	63 (47.4%)
	IV	10 (7.5%)
Coronary artery disease—*n* (%)	Yes	68 (53.1%)
Hypertension—*n* (%)	Yes	113 (89%)
Type 2 diabetes mellitus—*n* (%)	Yes	50 (41.7%)
Dyslipidaemia—*n* (%)	Yes	96 (78%)
Atrial fibrillation—*n* (%)	Yes	44 (37%)

Baseline characteristics and procedural information presented as median [25th–75th percentile] for continuous variables and count (%) for categorical variables.

### CT acquisition

CT imaging was performed on a GE 256 detector Revolution scanner (General Electric, Milwaukee, USA) or a Siemens 64 detector dual (Somatom Definition Flash, Siemens, Erlangen, Germany) both with a standard TAVR CT protocol (tube voltage 120 kV, tube current modulated from 150 to 725 mA based on patient size. Modes of the acquisition were assumed volume scan for GE revolution and helical for the Siemens Flash. Prospectively ECG gated non-contrast CT was performed in a sequential mode at 60–80% of the RR interval using a slice thickness of 3 mm. Contrast CT images were acquired using retrospective gating without tube current modulation of the entire cardiac cycle. Contrast injection rates depended on patients’ size, ranging from 4.5 mL/s to 6.5 mL/s with mean dose-length product of 1717 ± 496 mGy∗cm. Timing of contrast injection used bolus-tracking technique. A single bolus of contrast was administered to capture the cardiac structures and peripheral vasculature. The amount of contrast used was based on the patient’s estimated glomerular filtration rate (eGFR). Patients with an eGFR of <30 mL/min received 60 mL of contrast, whereas those with an eGFR of 30 mL/min or greater received 100 mL of contrast.

### Image analysis

**Figure 1:**
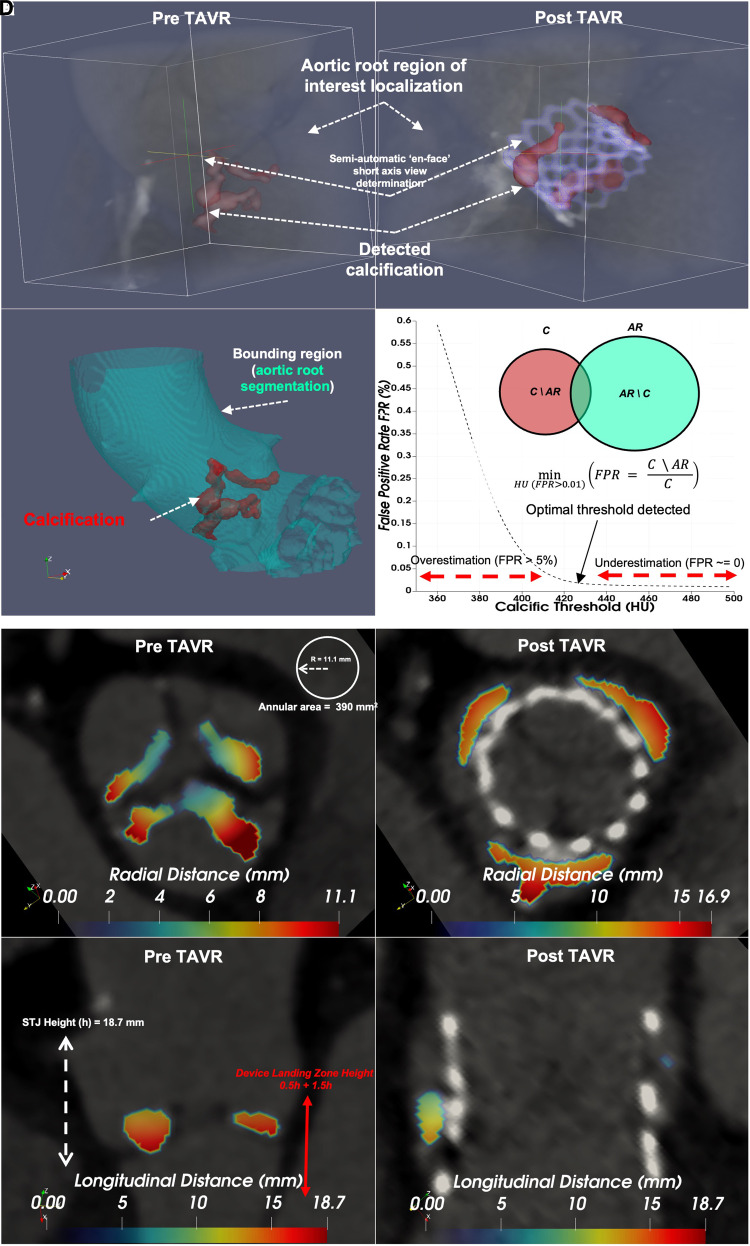
Visual representation of the computational framework developed for the assessment of calcification pattern and structure in contrast enhanced CT images for TAVR; (*A* and *B*) detected calcific segments are overlaid on the aortic root segmentation and device stent (outlined) before and after TAVR; (*C* and *D*) the FPR method detects calcification by optimizing the false positive rate between aortic root lumen and calcific segments (outlined); (*E* and *F*) the 2D ‘en-face’ short axial view near the annular plane shows calcification distances to the annular centre in a range from 0 to the annular radius; (*G* and *H*) the 2D long-axis view before and after TAVR shows calcification distances to the annular plane in a range from 0 to the height of the sinotubular junction (STJ).

**Figure 2:**
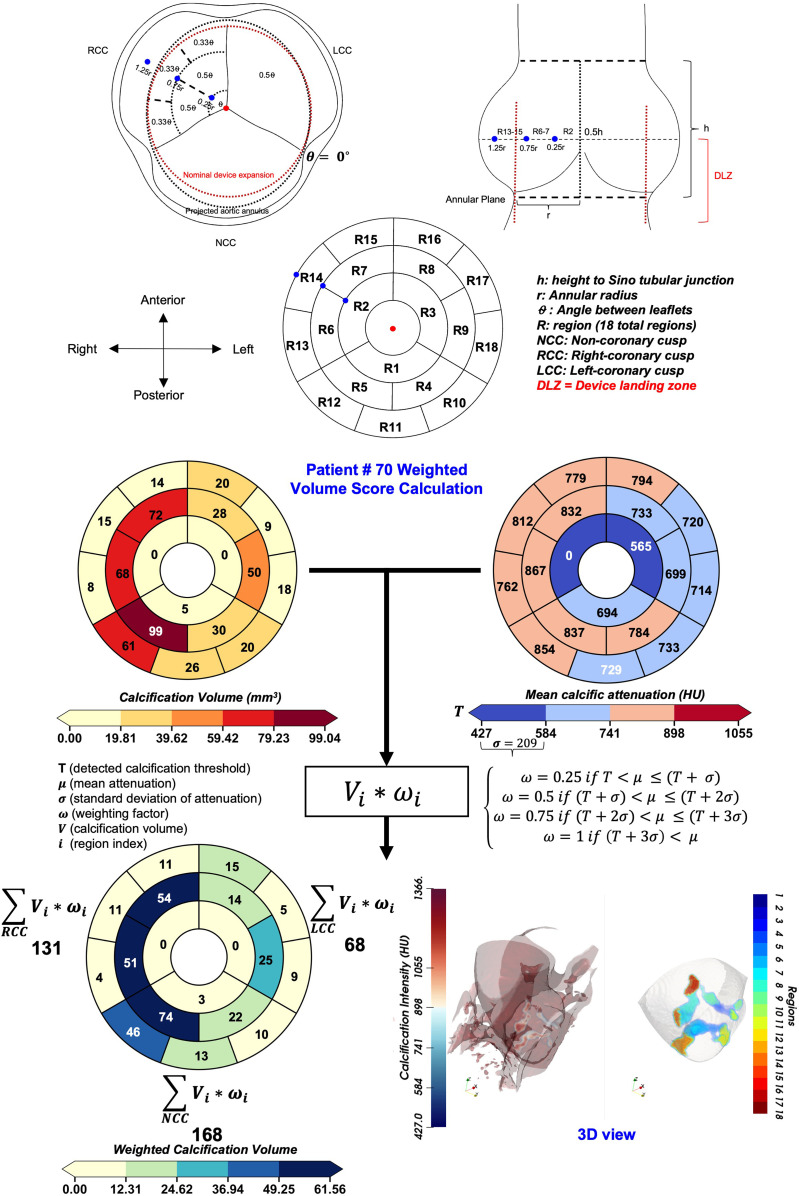
An 18-region cylindrical coordinate map is used to model the regional contribution of different regions in the landing zone. Region subdivisions (R1-R18) are based on parametric coordinates in cylindrical coordinate system, normalized by patient specific aortic root dimensional measurements (STJ height **h**, annular area derived radius **r**, and angles between the interleaflet edges measured from the ‘en-face’ short-axis view of the valve). An example patient calculation is shown with the derived volume and mean HU intensity bullseye maps. The final value in each region is calculated as a multiplication of volume and an intensity weighting factor; which is based on the location of the intensity value on a scale between minimum calcific threshold and 4 ∗ standard deviation of HU intensity in the entire calcific volume. Finally, the summation is calculated in the six regions associated with each cusp to generate individualized intensity weighted scores for each patient.

Previously, we developed a new method that semi-automatically finds a threshold to minimize the FPR,^14^ defined as the ratio of falsely labelled calcific pixels to the total number of calcific pixels (*Figure 1A*–*D*). We introduce a new scheme for describing the geometric and relative concentration of calcification via intensity as well as radial and longitudinal maps (*Figure 1E*–*H*). Our computational framework requires the field of view to initially be localized to the aortic valve based on the standard assessment guidelines for TAVR planning (*Figure 1A*). Subsequently, we generate density and topographic maps of both radial and longitudinal measurements for the detected calcific regions (*Figure 1E*–*H*). Finally, we developed an anatomical region mapping scheme (*Figure 2*) based on standardized measurements of height to sinotubular junction, annular area derived radius, and angles of the inter-cusp triangle. These measurements were used as relative distance thresholds to quantify regional calcific volume and average Hounsfield intensity for each patient (*Figure 2*). Based on each patient’s parametric map, an intensity-weighted volume score was calculated for reach cusp in the device landing zone, the regional mapping scheme, and an example calculation for a sample patient is presented in (*Figure 2*).

### Evaluation parameters

The annular area and height of sinotubular junction (STJ) were measured as per TAVR assessment guidelines. The ‘area cover index’ represents that the percentage of oversizing of the THV as compared with the measured annulus size is calculated using the formula [(nominal THV area − measured annular area)/nominal THV area] ∗ 100%. All dimensional measurements were performed in mid-systole. The standard ‘Agatston score’ was measured using pre-TAVI non-contrast CT images using calcium scoring application (Syngo.via; Siemens Healtheneers). Finally, the qualitative ‘calcification grade’ was assessed visually based on the following criteria (mild: non-protruding calcific lesions on one or more cusps, with minimal extension into the annular lumen; moderate: calcific lesions with some extension into the annular lumen and/or some protrusion into the LVOT; severe: multiple or single calcific nodules with clear extension into the annular lumen and/or protrusion into the LVOT). All the latter measurements were performed independently by the local heart team(s). The ‘device landing zone calcification’ (DLZ) was determined from a region of interest covering the entirety of the aortic root using the FPR method at late-diastole. All calcification in the aortic valve complex was separated automatically in the craniocaudal axis along the double oblique long-axis of the LVOT/aortic valve into the following regions: leaflet (from 0.5* height of STJ to the annular plane), annular/LVOT (from annular plane to 1.5* height of STJ); and device landing zone (DLZ) (encompassing all of the above). Volume of calcification (CA) (in mm^3^) was recorded for each region (DLZ calcific volume, ‘leaflet calcification volume’, and ‘annular/LVOT calcification volume’). Finally, the DLZ calcific volume was divided into six sectors for each cusp as follows: non-coronary, left, and right (NCC, LCC, and RCC), respectively, with three radial regions and angular divisions (from 1 to 3) increasing towards the edge of the leaflets. The volume in each volumetric area is recorded and then multiplied by an intensity weighting factor (0.25–1) based on the mean HU attenuation of region and the standard deviation of the entire DLZ calcific volume. The total score for each cusp is then calculated as the sum of the intensity-weighted volumes giving the final ‘DLZ intensity-weighted calcification volume’ for the NCC, RCC, and LCC, respectively (*Figure 2*). For the sub-cohort with post-TAVR CT imaging available, the expanded valve diameter was measured at three different longitudinal views at the cusp plane. The average diameter was calculated and used to estimate the expanded valve area after implantation based on circular area assumption. The percentage change between nominal and expanded valve area after implantation was used to calculate the ‘Deviation from nominal expansion (%)’ that was used as a dependent variable to assess the effect of evaluated parameters on valve expansion behaviour.

### Statistical analysis

**Figure 3:**
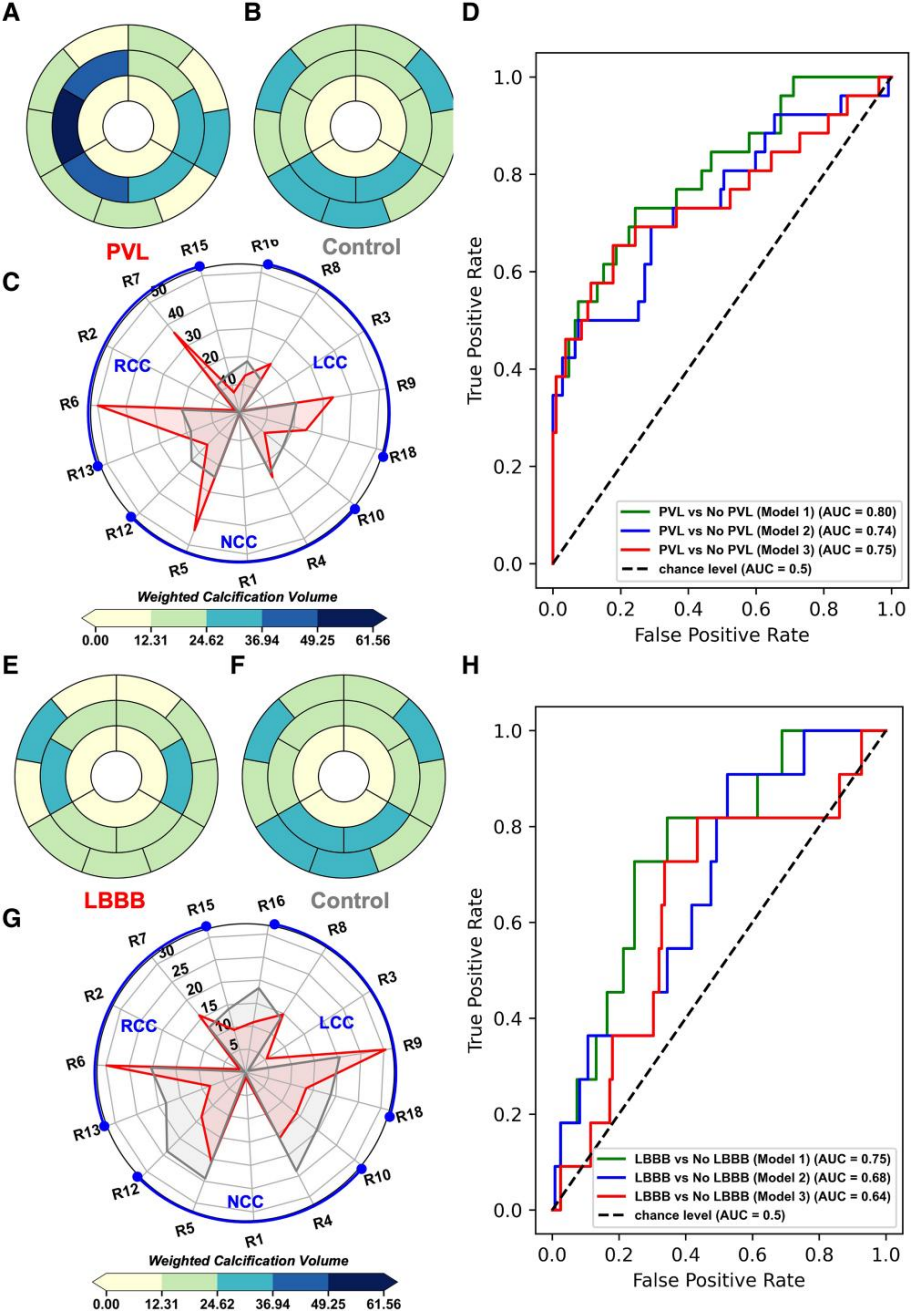
Landing zone calcific distribution in post–procedural complications; (*A* and *B*; *E* and *F*) bullseye landing zone weighted calcification volume median bullseye plots comparing outcome group vs. control. (*C*; *G*) Radar plot with categories comparing outcome group vs. control in terms of regional median volume spread in each region. (*D*; *H*) ROC curve analysis for multinomial multivariate logistic regression models in terms of each outcome vs. control, the nested logistic regression models (Models 1–3) parameters are presented in *Table 2*.

**Figure 4:**
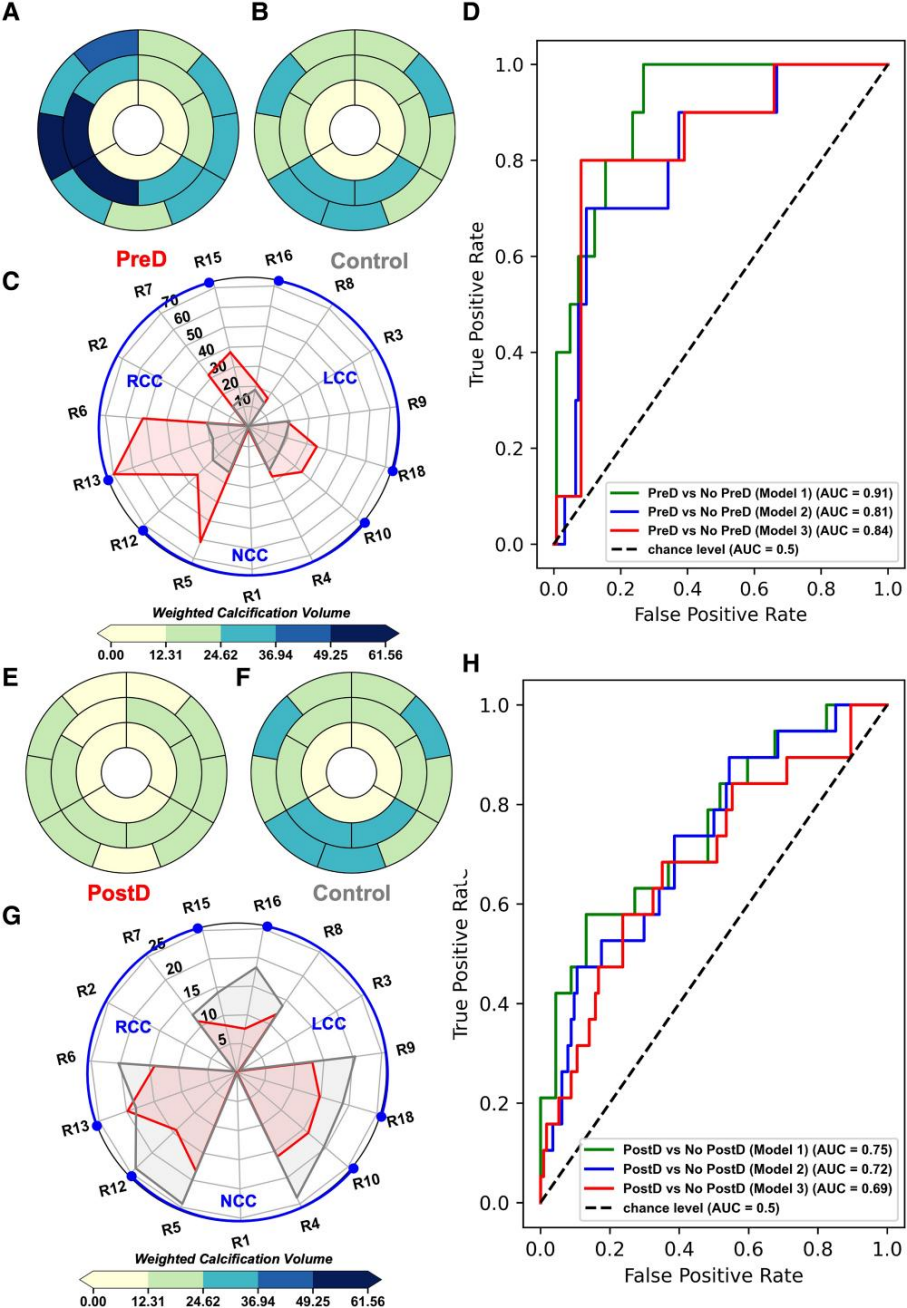
Landing zone calcific distribution in peri-procedural events; (*A* and *B*; *E* and *F*) bullseye landing zone weighted calcification volume median bullseye plots comparing event group vs. control. (*C*; *G*) Radar plot with categories comparing event group vs. control in terms of regional median volume spread in each region. (*D*; *H*) ROC curve analysis for multinomial multivariate logistic regression models in terms of each event vs. control, the nested logistic regression models (Models 1-3) parameters are presented in *Table 2*.

Regression analysis and modelling were performed using Jamovi v.1.8 and Python scikit-learn (v.1.1). Summaries of the variables and tests performed are outlined as follows. Multinomial multivariate logistic regression was used to evaluate a multi-class classification model using a nested model with incremental addition of dependent parameters starting with baseline parameters (Model 3: calcification grade, Agatston score, valve size, and area cover index) then adding (Model 2: leaflet and ALVOT calcification volume) and finally adding (Model 1: DLZ intensity-weighted calcification volumes NCC, RCC, and LCC). Comparison between the models was based on likelihood ratio tests (LRT), where the delta chi-squared statistic ($\nabla \chi^{2}$) quantifies the improvement in event/outcome prediction over the reduced models with less parameters. Individual parameter effects were also assessed using the LRT test with *P* < 0.05 indicating significant improvement in explaining the variance in the data. Finally, receiver operating characteristic (ROC) curves were used to evaluate the classification power of each model in both multivariate (*Figure 3D*–*H*; *Figure 4D*–*H*; *Table 2*) and univariate schemes (see Supplementary data online, *Figure S1A*–*D*; Supplementary data online, *Table S1*), by calculating the area under the curve (AUC), which was interpreted as follows: 0.5–0.6: fail; 0.6–0.7: poor; 0.7–0.8: acceptable; and 0.8–0.9: excellent. For the sub-cohort with post-CT imaging, a multivariate linear regression model was fitted with % change between measured expanded valve area and nominal area of valve before intervention as a dependent variable (see Supplementary data online, *Figure S1E*; Supplementary data online, *Table S2*). Finally, non-parametric one-way analysis of variance (ANOVA) was used to evaluate the differences between the proposed intensity-weighted regional volume scores and qualitative calcification grades followed by Dwass-Steel-Critchlow-Fligner *post hoc* comparisons (see Supplementary data online, *Figure S1F*). Aggregate summary plots for intensity-weighted volume scores are presented in Supplementary data online, *Figure S2*. Finally, interobserver variability for the calcium detection method was measured using the intraclass correlation coefficient (ICC; two-way random agreement). All evaluated parameters were reported as median [25th–75th percentile] or counts (%) for categorical variables, with summary statistics presented in Supplementary data online, *Table S1*. For all tests, statistical significance was considered when the *P*-value was <0.05.

**Table 2:** Summary of multivariate logistic regression results

Multinomial logistic regression*_n_*_= 133_	Event/outcome	Model 1			Model 2		Model 3			
		DLZ intensity-weighted calcification volume			Calcification volume		Conventional clinical parameters			
Statistical tests		NCC	RCC	LCC	Leaflet	ALVOT	Calcification grade	Agatston score	Valve size	Area cover index
AUC	PVL	0.8 (*P* < 0.001)			0.744 (*P* < 0.001)		0.751 (*P* < 0.001)			
	LBBB	0.748 (*P* < 0.001)			0.679 (*P* < 0.05)		0.636 (*P* < 0.05)			
	PreD	0.907 (*P* < 0.001)			0.811 (*P* < 0.001)		0.837 (*P* < 0.001)			
	PostD	0.75 (*P* < 0.001)			0.724 (*P* < 0.001)		0.685 (*P* < 0.001)			
Sensitivity/specificity (cut-off)^a^	PVL	73.1/72.9 (0.22)			69.2/69.2 (0.21)		69.2/69.2 (0.19)			
	LBBB	72.7/73 (0.09)			54.6/58.2 (0.07)		63.6/66.3 (0.09)			
	PreD	80/79.6 (0.08)			70/69.9 (0.06)		80/79.6 (0.07)			
	PostD	63.2/63.2 (0.13)			63.2/63.2 (0.15)		63.2/64.9 (0.17)			
Parameter comparison^b^ $(\chi^{2},\;P\text{-}\mathrm{value}$)	Model 1	7.62 (ns)	18.1 (*P* = 0.001)	8.49 (ns)	20.3 (*P* < 0.001)	5.3 (ns)	18.1 (*P* = 0.001)	21.36 (*P* < 0.001)	10.74 (*P* < 0.05)	11.7 (*P* < 0.05)
	Model 2				11.8 (*P* < 0.05)	6.85 (ns)	14.5 (*P* < 0.01)	12.7 (*P* < 0.05)	9.4 (ns)	13.2 (*P* = 0.01)
	Model 3						17 (*P* < 0.01)	14.5 (*P* < 0.01)	6.9 (ns)	11.1 (*P* < 0.05)
Model comparison^c^ $(\nabla \chi^{2},\;P\text{-}\mathrm{value}$)	Model 1 vs. Model 2	18.4 (*P* < 0.05)								
	Model 1 vs. Model 3	50.9 (*P* < 0.001)								
	Model 2 vs. Model 3	32.5 (*P* = 0.001)								

Tabulated summary of parameters used in multinomial multivariate logistic regression using nested Models 1–3.

a Cut-off thresholds for ROC curves were based on maximizing sensitivity and specificity.

b Chi-square statistic presented for each parameter within the nested model.

c Change in χ^2^ statistic for overall evaluation of incremental addition of parameters from the baseline (Model 3).

## Results

We quantified the pattern and structure of DLZ calcification in terms of calcific volume and an intensity weighting factor based on average HU intensity in each region (*Figure 2*). The analysis of patterns was carried out on patients reported with at least one of the following events and/or outcomes: PVL ≥ mild: *n* = 26/133 (14.3%); new LBBB: *n* = 11/136 (8.3%); preD: *n* = 10/133 (7.5%); and postD: *n* = 19/133 (18.3%). A control group was used for comparison defined as patients who had none of the latter events/complications [control: *n* = 67/133 (50%)]. In addition, leaflet calcification volume, annular/LVOT calcification volume, the clinically assessed calcification grade, Agatston score, area cover index, and valve size were included in the regression analysis. Interobserver variability for detected total calcific volume was assessed on a random sub-group of 49 patients using intraclass correlation coefficient (ICC; two-way random agreement) showing excellent reproducibility (ICC: 0.95; 95% CI 0.94–0.96; raters = 2).

### Calcification and procedural outcomes

We analysed the 18-region intensity-weighted volume maps for PVL vs. the control group (*Figure 3A*–*C*; Supplementary data online, *Figure S2A* and *B*). We observed more calcific deposition in the RCC regions of the landing zone compared with control. In the control group, calcification patterns were more symmetric, with PVL patients having greater absolute difference between adjacent regions. Finally, ROC curve analysis with a model that includes the new indices showed excellent classification power (AUC = 0.8, sensitivity = 73.1%, specificity = 72.9%) (*Figure 3D*). In terms of model comparison (*Table 2*), the inclusion of leaflet or ALVOT calcific volume did not improve classification power vs. a baseline model using qualitative calcification grade (AUC = 0.744 vs. AUC = 0.751). In univariate analysis, area cover index showed the best classification power with an acceptable AUC = 0.73, sensitivity = 65.4%, and specificity = 64.5%. For the LBBB group vs. the control (*Figure 3E*–*G*; Supplementary data online, *Figure S2C* and *D*), calcific deposition was generally lower compared with control, especially in the RCC, and overall symmetry was lower compared with control towards the root attachment. ROC curve analysis with a model that includes the new indices showed acceptable classification power (AUC = 0.748, sensitivity = 72.7%, specificity = 73%) (*Figure 3H*). In terms of model comparison (*Table 2*), both alternative models without the intensity-weighted scores had poor classification ability (AUC = 0.679 and AUC = 0.636). None of the parameters were significant in predicting LBBB in terms of univariate analysis.

### Calcification and device dilation

Patients who had pre-dilation prior to direct expansion had relatively higher calcific deposition over most regions compared with control (*Figure 4A*–*C*; Supplementary data online, *Figure S2E* and *F*); with high concentrations of calcium towards the right and left cusps with clear asymmetry in calcific deposition. In terms of classification ability, the model with the intensity-weighted scores had the highest classification ability compared with total volumes or calcification grade alone (AUC = 0.907, sensitivity = 80%, specificity = 79.6%). In terms of model comparison (*Table 2*), all models had excellent discriminative ability in terms of deciding pre-dilation (AUC = 0.811; AUC = 0.837). In univariate analysis, the weighted RCC volume score and Agatston score showed significant classification power with acceptable AUC = 0.74 and AUC = 0.83, respectively; conversely, for patients who underwent post-dilation of the device (*Figure 4E*–*G*; Supplementary data online, *Figure S2G* and *H*). Calcific deposition was markedly similar to control, with relatively lower calcific deposition in regions near the root attachment. All multivariate models had relatively similar acceptable classification ability with AUC = 0.75, AUC = 0.724, and AUC = 0.685 for Models 1, 2, and 3, respectively (*Table 2*). In univariate analysis, the weighted NCC volume score showed significant classification power with a poor AUC = 0.69, sensitivity = 57.9%, and specificity = 60%.

### Calcification and device expansion

Finally, in terms of the parameters effect on % deviation from nominal area after implantation, a nested multivariate linear regression model was fitted on a small sub-cohort (*n* = 33) with post-CT imaging available (see Supplementary data online, *Figure S1E*; Supplementary data online, *Figure S3*). The complete nested model with all parameters used only accounted for 56% of the variance in the change, with no significant *r*-squared differences between the incremental models. Only the valve size and calcification grade appeared as significant predictors in the multivariate linear regression (Supplementary data online, *Table S2*).

### Incremental parameter effects

In terms of parameter effect in the nested logistic regression model, LRT^16^ were used to evaluate and compare the predictor variables (*Table 2*). Significant predictors ordered by strongest to weakest in terms of χ^2^ are listed as follows: Agatston score, leaflet calcification volume, calcification grade, DLZ intensity-weighted calcific volume RCC, qualitative calcification grade, area cover index, and valve size. Overall, nested model difference showed significant changes with a delta χ^2^ change of ($\nabla \chi^{2}$ = 18.1; *P* < 0.05), ($\nabla \chi^{2}$ = 50.9; *P* < 0.001), and ($\nabla \chi^{2}$ = 32.5; *P* < 0.001) between Models 1–2, Models 1–3, and Models 2–3, respectively, indicating that the overall fit of the prognostic model for all event/outcome is better satisfied with the model (‘Model 1’) that also includes the DLZ intensity-weighted calcification volume parameters. The clinical validity of the proposed intensity-weighted volume scores is further confirmed through a one-way non-parametric ANOVA analysis. This analysis revealed significant concordance between the proposed intensity-weighted volume scores and clinically evaluated qualitative calcification grades (see Supplementary data online, *Figure S1F*), with *post hoc* tests showing significant changes between severe and none for all regions and for RCC and LCC between moderate and none grades.

## Discussion

Improved TAVR valve designs, increased operator experience, and peri-procedural planning with multimodality imaging have greatly reduced risks associated with TAVR.^4^ Accurate annular sizing is a robust way to reduce the risk of PVL and conduction abnormalities, and pre-procedural sizing has been shown to reduce risks of mortality and morbidity.^4,13^ Similarly, determination of the calcium burden and distribution pattern in the AV and left ventricular outflow tract (LVOT) are predictors for PVL, annular rupture, and the need for a second valve implantation.^7,11,17^ The relationship between post-TAVR complications and TAVR risk assessment both with or without pre- or post-dilatation is ambiguous.^10^ For example, the presence of severe valvular calcium is a mechanical barrier to the ideal device expansion, and it might seem appropriate to perform a pre-dilation to improve subsequent implantation. However, this approach mediates non-circular valve expansion.^10^ Alternatively, post-dilatation is commonly used to correct for sub-optimal outcomes immediately following the deployment of the device. However, this may contribute to long-term prosthetic cusp damage that contributes to valve degeneration^10,18–20^ and further increases risk of new conduction abnormalties.^10,18^ In our opinion, selecting the best course of action can be more confidently accomplished by carefully examining the unique 3D morphology and intensity distribution of the landing zone calcification as proxy for tissue density. The mechanical interaction between the deforming stent under the balloon pressure and dense calcium deposits anchored around the device landing zone may interfere with optimal final configuration.^18,21,22^ Even though the total calcification volume is a well-studied parameter,^23,24^ it is insufficient to predict how the calcification will interact with the stent without taking the location and relative density into account.

### Intensity-weighted volume scoring showed a significant improvement for predicting PVL and LBBB over current predictive models

We showed that patients diagnosed with PVL greater than or equal mild following TAVR had more calcific depositions closer to the annular centre with a higher relative density compared with the control (*Figure 3A*–*C*). The pronounced asymmetry towards the non- and right coronary cusps combined with lower relative calcification in the left coronary cusp may explain genesis of PVL due to reduced sealing between the device skirt and the aortic annulus. Furthermore, we highlight the natural asymmetry of valve morphology that corresponds with the inner curvature of the ascending aorta that coincides with the side of the left coronary cusp, which was found to be a major site of paravalvular leaks in previous investigations.^7,11,25^ The presence of this pattern had excellent classification power in differentiating risk of PVL from the control based on ROC curve analysis (*Figure 3D*). Quantification of such patterns can help decide on modifiable interventional factors such as valve size, degree of expansion, dilation strategy, and positioning.^3,6,10^

In contrast, patients who were diagnosed with new LBBB following TAVR had relatively lower calcific deposition especially in regions associated with the RCC compared with control (*Figure 3E*–*G*). This deviation from the control pattern in combination with inherent asymmetry between left and non-coronary cusp regions might explain incidence of LBBB due to increased radial force imposed by the stent on the NCC–RCC side of the ventricular outflow tract.^8^ This region is near a left ventricular bundle branch, and previous studies showed asymmetry in this region corresponding with higher rates of subsequent pacemaker implantation.^3,8^ In addition, annular calcification in the RCC was also found to be an important predictor of new LBBB following TAVR.^9^ The proposed model that included the regional intensity-weighted volume scores greatly increased the predictive power of the model vs. models that did not include these indices, which were deemed as poor based on ROC curve analysis (*Figure 3H*). As LBBB is one the most common risk factors associated with TAVR and may require permanent pacemaker implantation post-TAVR, an ability to recognize patients with higher risk of developing this serious complication may help the treating physician in terms implantation height and percentage of oversizing.

### Intensity-weighted volume scoring showed an incremental improvement for predicting need for pre- or post-dilation vs. conventional decision models

Both pre- and post-dilation aim to reduce the risk of peri-procedural complications, while maximizing the area of blood flow.^4,10^ Cost vs. benefit analysis of both techniques is challenging due to limited parameter quantifying regional calcific burden.^10^ In that respect, the proposed combined intensity and volume mapping may be especially important in stratifying risk factors associated with both techniques. In our analysis, we found that patients who received pre-dilation had significantly higher intensity-weighted volumes across most regions in the landing zone compared with control (*Figure 4A*–*C*). Furthermore, the combined model that includes the regional intensity scores greatly improved classification power compared with alternative models based on ROC curve analysis (*Figure 4D*). The distribution of the calcifications pattern for pre-dilation group implies a highly stenotic valve that may restrict optimal device expansion.^10^

Alternatively, patients who had post-dilation seem to have patterns that were lower compared with control (*Figure 4E*–*G*). Although, the addition of the proposed indices modestly improved the classification ability compared with the simpler models (*Figure 4H*), we should emphasize the importance of considering intensity weighting as a proxy for calcific density; since the local calcific density cannot be estimated from visual inspection of CCTA scans. We propose a quantitative measure of the density based on the regional mapping scheme. Establishing quantitative thresholds to detect patterns that indicate a high inter-cusp calcific asymmetry may provide a predictor for paravalvular leakage near diffuse calcific regions^10,23^ and hence an important add-on for pre-operative planning using CCTA.

### Effect of calcification patterns on device expansion

While the use of CCTA pre-TAVR for planning and risk stratification is widely used, the role of CT imaging post-TAVR is not yet clear. Usually, post-TAVR CCTA is utilized when major complications, such as coronary occlusion, aortic rupture, or bleeding, are suspected, and the role of routine post-TAVR CCTA is controversial.^6^ Post-TAVR CCTA can be used to assess the expansion, eccentricity, and position of the valve.^6^ Limited information exists on the biomechanical interaction between the device during expansion with adjacent structures including calcific lesions in the landing zone.^18,21^ We have assessed the multivariate nested models on a sub-cohort of 33 patients to determine the effect of the parameters on measured vs. nominal valve area in a linear regression scheme (see Supplementary data online, *Table S2*). Overall, the models had modest predictive power with only the valve size and area cover index that were found to be significantly associated with difference in device area after implantation (see Supplementary data online, *Table S2*). This is indicative that calcification patterns primarily play a role in adjudicating clinical decision in device selection, sizing, expansion rate, positioning, and dilation strategy, especially in balloon-expandable devices. Further analysis using computer-aided simulations^17,18,20,21,25^ or *in vitro* experiments^22^ could help shed light on the interaction between these factors.

### Incremental value and application of proposed metrics over conventional parameters

**Figure 5:**
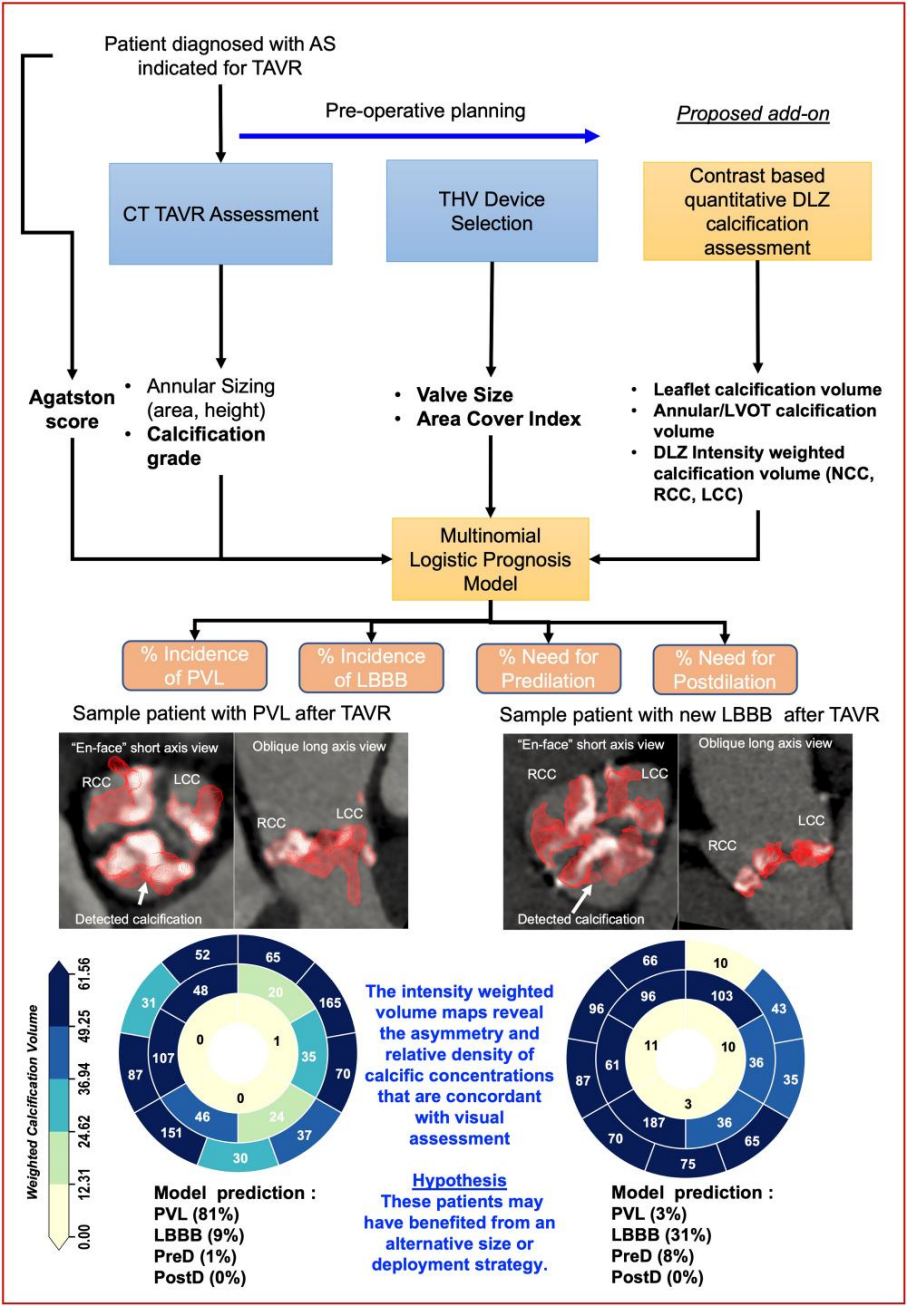
Typical TAVR pre–operative planning workflow with appended proposed add–on. The proposed add–on complements the standard CCTA acquisitions by quantifying device landing zone characteristics. The set of parameters from the device landing zone in addition to existing clinically assessed parameters can then be used as input to a prognostic model that can predict incidence probabilities of events/outcomes (e.g. PVL/LBBB) simultaneously.

Currently, there is no standardized method for quantification of DLZ calcific burden.^2,13^ The conventional Agatston score requires non-contrast CT acquisitions with 2.5–3 mm axial resolution and requires using axial views to reproduce thresholds for severity determination that does not allow assessment of native valve dimensions and is limited to leaflet/cusp calcification.^2,13^ These requirements preclude regional calcific assessment and are not well defined for prognostic purposes. CCTA in TAVR offers superior spatial and contrast resolution. However, due to variability in contrast absorption, there as of yet no standard method for quantification of calcification using these types of images^2,13^ and assessment of calcific burden is mainly done through visual assessment. Furthermore, regional quantification is complicated by interobserver variability, natural variance in valve anatomy as well as physiological changes due to disease remodeling.^23,26^ Our proposed framework aims to address these challenges while complementing the standard pre-operative planning stage of CT-TAVR. First, by automatic detection of calcification in DLZ. Secondly, we introduced a patient specific regional description that is designed to capture geometrical variation in calcific deposition and automatically separate the anatomical regions of interest. Thirdly, we introduce an intensity weighting factor that is adapted to contrast images, which can capture structural variation in calcific deposition by taking into account variations in the density of calcification based on local intensity variations. We showed that the more detailed information obtained through intensity-weighted volume scoring may lead to better risk stratification through LRT and ROC curve analysis. Intensity-weighted volume scoring goes beyond visual assessment or bulk quantification of calcification volume. Combined with a multinomial prognostic model, the framework aims to extend the standard pre-operative planning steps with quantitative metrics optimized for common TAVR complications and/or deployment decisions, without incurring additional time or resource costs (*Figure 5*). Finally, the design of the maps and intensity weights may provide future benefit by optimizing the intensity weights to improve classification accuracy on larger datasets through advanced machine learning and personalized computational modelling techniques.^27–30^

## Limitations

This study has several limitations. First, as our study is an analysis of a modestly sized observational cohort, unavailable procedural confounders such as volume of balloon pre-/post-dilation, initial device landing zone position, device angle at deployment, and incidental observations with peri-procedural echo or fluoroscopy might have influenced the observed findings. Thus, the overall findings in our study should be considered exploratory and hypothesis-generating only. Secondly, given the relatively small sample size of patients and clinical events, our study might be underpowered in terms of multivariate analysis robustness. Thirdly, the current study was only performed for tricuspid patients who underwent TAVR with balloon-expandable devices. Finally, the study included some patients who underwent the procedure with an older Sapien XT valve device. Further investigation is needed in a larger randomized multi-centre trial to better judge the effectiveness of the method in more clinical contexts as well as interscan and inter-device settings.

## Conclusions

Despite the evidence for the role of calcification patterns in prognosing different outcomes of TAVR, the role of relative calcific density and its causal effect on valve expansion is limited. We developed a computational tool designed for detailed quantification of landing zone calcific location, quantity, and relative density using routinely used CCTA imaging prescribed for TAVR planning. These new markers could be particularly important, as an add-on to exiting CT-TAVR guidance protocols given that in current CT-TAVR assessment, calcification severity is already confirmed, and valvular calcification is described qualitatively to plan the procedure. The presented computational tool for calcification burden in the aortic valve using CT images can be potentially used as follows: (i) a complementary method to existing TAVR workups, providing a way to differentiate qualitative degrees of landing zone calcification severity, which may lead to better evaluations of procedural techniques (pre- and post-dilation) and/or optimal device type and sizing; and (2) a tool for predicting patients with higher risk of PVL and LBBB. Our preliminary model benefits from considering the interaction between these factors in a combined multi-class predictive scheme that is especially important due to the complex interplay of predictor parameters in the interventional setting.

## Supplementary Material

qyad027_Supplementary_Data

## Data Availability

Not applicable.

## Lead author biography

Mohamed Abdelkhalek is currently a PhD candidate in the biomedical engineering department in McMaster University, Canada in MotamedLab. He received the B.Sc. degree in computer science and engineering from German University in Cairo, Egypt and the M.Sc. degree in robotics control and smart systems from American University in Cairo, Egypt. His focus is on computational modelling of cardiovascular diseases with a focus on the development of computational platforms for interventional planning and assessment. He has authored and co-authored works in JACC: Cardiovascular Imaging, Structural Heart, and IEEE transactions in Biomedical Engineering as well as conference publications in JCCT, ESC, and AHA.
