# Supplementary material for: Incremental prognostic value of intensity-weighted regional calcification scoring using contrast CT imaging in TAVR
Source: Eur Heart J Imaging Methods Pract. 2023 Sep 29;1(2):qyad027. doi: 10.1093/ehjimp/qyad027 (PMC11195730; doi:10.1093/ehjimp/qyad027)
Supplement: qyad027_Supplementary_Data [file qyad027_Supplementary_Data.zip › SupplementaryFiles.docx]

List of Supplementary figures and tables

**Fig. 1. Additional univariate and linear regression analysis of predictors. (a-d)** Univariate ROC curve analysis for multinomial logistic regression models in terms of each outcome/event versus control. **(e)** Box plots comparing cusp specific intensity weighted calcification volume across clinically evaluated qualitative calcification grades, results of one-way ANOVA parametric analysis annotated to compare between grades. **(f)** Scatter plot showing linear regression results of models 1-3 on a sub cohort of 33 patients with post-CT imaging comparing predicted versus measured percentage change of nominal area after TAVR

**Fig. 2. Summary comparison for DLZ intensity weighted calcific regions between event/outcome groups and control. (a;c;e;g)** Bar plot comparing event/outcome group vs control in terms of regional contribution of the different landing zone regions **(b;d;f;h)** Box and whisker plot comparing total landing zone weighted calcific volume between event/outcome group vs control

**Fig. 3. Total and regional of calcification in pre vs post TAVR (representative cases). (a-d)** Six representative patients with pre and post CT images from each of the following groups was selected: Paravalvular Leakage **PVL**, left-bundle-branch-block **LBBB**, pre-dilation **PreD,** post-dilation **PostD. (e-f)** two control cases. Annotated values show measured values for parameters used in regression analysis listed as follows: **wv_ncc_, wv_rcc_**_,_ **wv_lcc_ :** regional intensity weighted volume scores. **Lcv, Alvotv, Aci, Ag, Cg:** leaflet calcification volume, annular/lvot calcification volume, area cover index, Agatston score and calcification grade. **Ds, Ed, Dna, Ei:** device size, expanded valve diameter, percentage deviation from nominal expansion, eccentricity index.

**Table. 1. Summarized statistics (N=133).** Grouped by PVL: Paravalvular leakage, LBBB: Left-bundle-branch-block, PreD: Baloon pre-dilation and PostD: Baloon post-dilation. Parameter values presented as median [25^th^ – 75^th^ percentile] for each subgroup. Area under curve (AUC) and significance p-values reported for significant predictors (p < .05) under univariate logistic regression analysis.

**Table. 2. Summary of multivariate linear regression results.**  Tabulated summary of parameters used in multivariate linear regression using nested models 1-3 to evaluate effect on deviation from nominal expansion DNA (%). The F-score and root mean square error (RMSE) represent the overall accuracy and strength of the predictor variables and models. *F-score statistic presented for each parameter within the nested model representing it’s influence on the overall model(s). ** change in r-square statistic for overall evaluation of incremental addition of parameters from the baseline (Model 3).


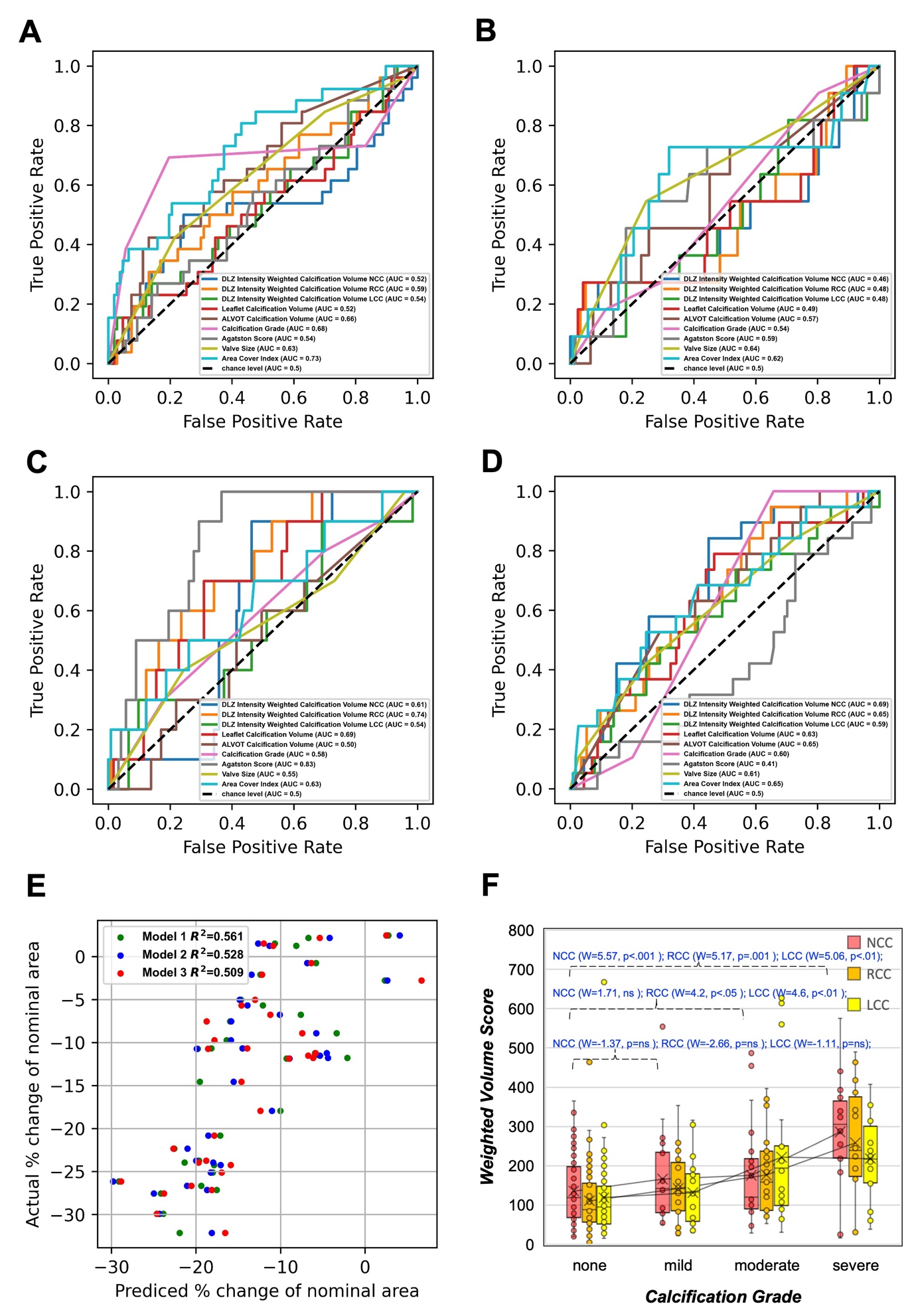


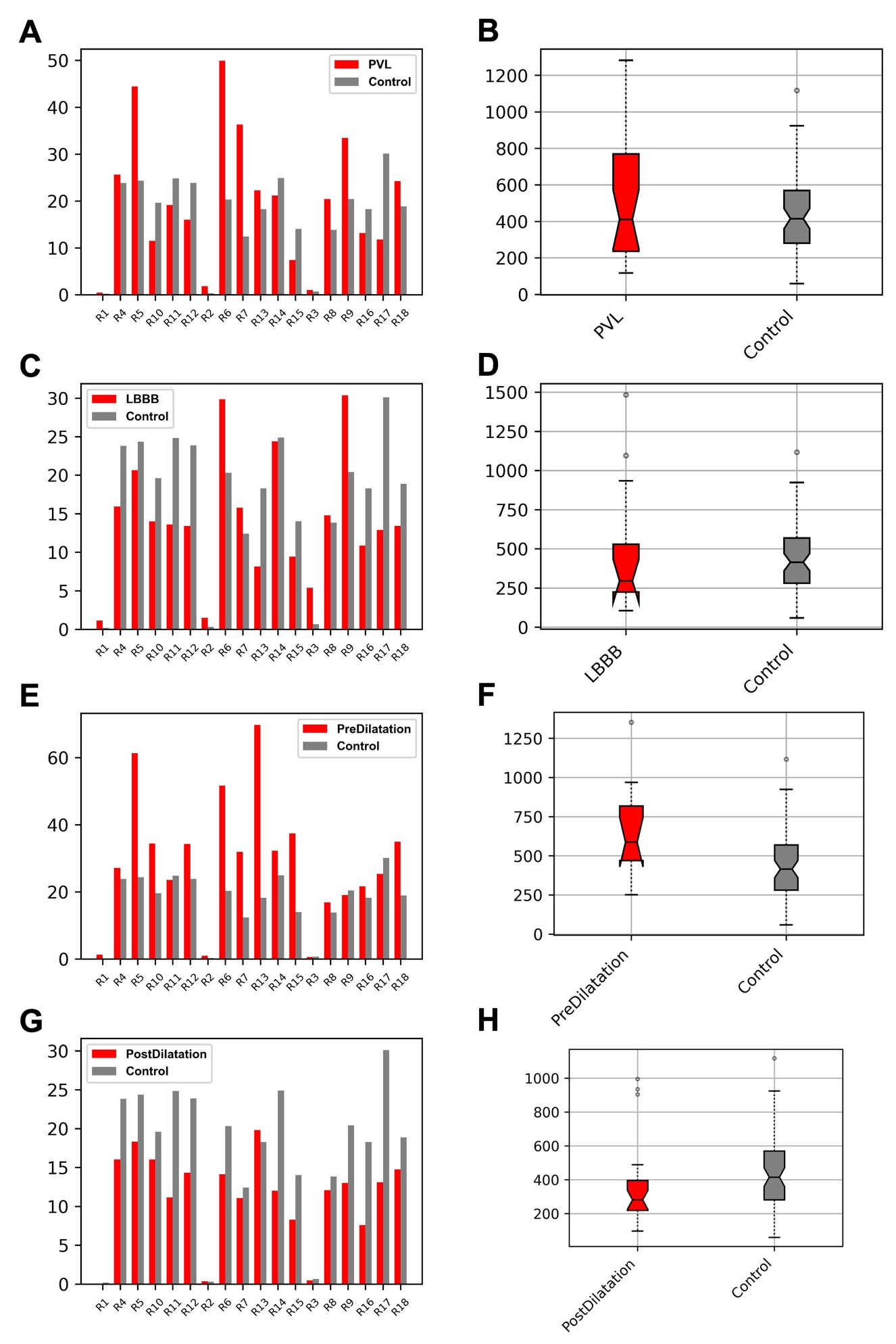


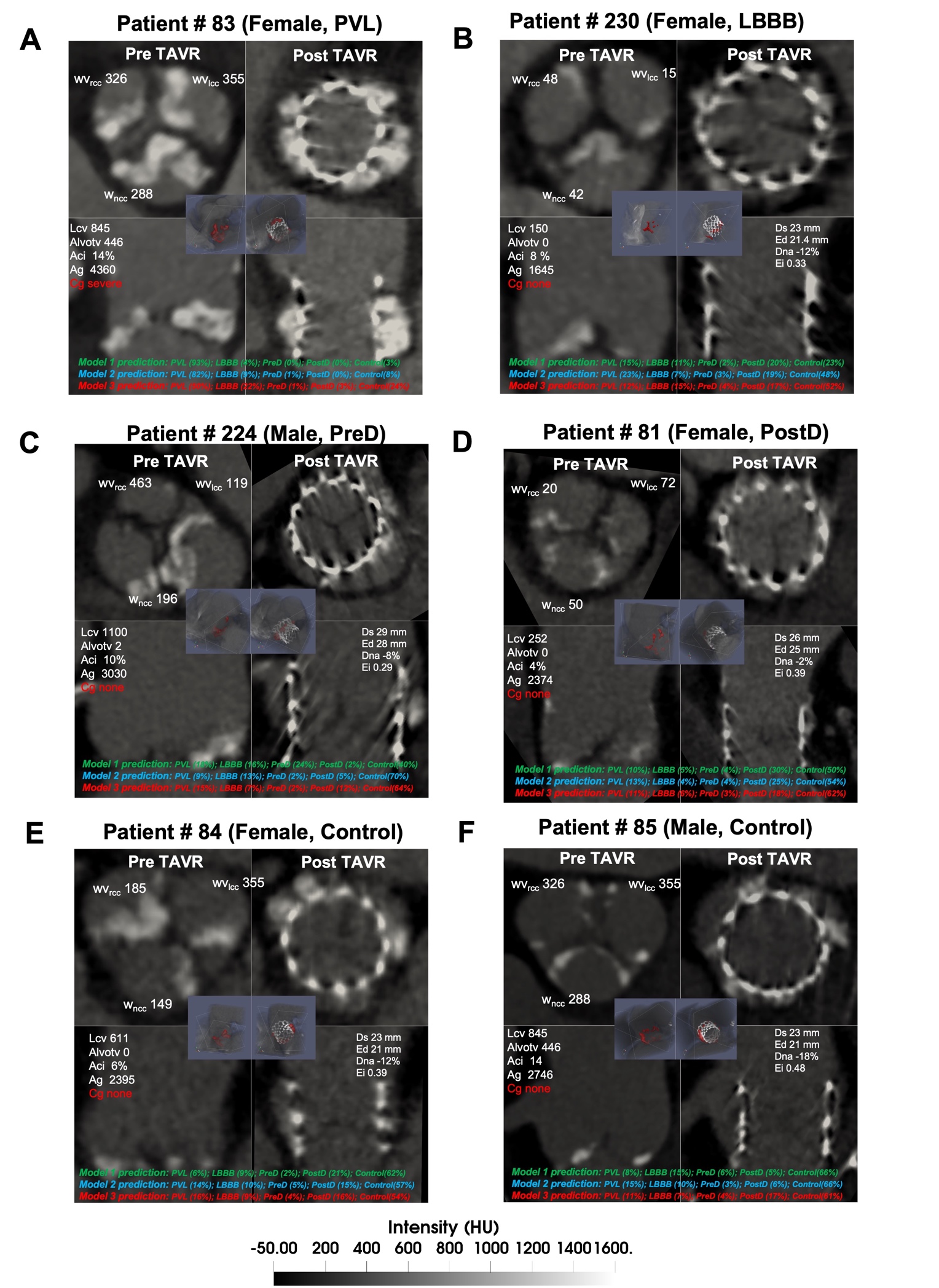


| **Parameter** | **PVL_(n=26)_** | **LBBB_(n=11)_** | **PreD_(n=10)_** | **PostD _(n=19)_** | **Control_(n=67)_** |
| --- | --- | --- | --- | --- | --- |
| **Device landing zone calcific weighted volume - NCC** | **196[66-276]**  AUC=0.52 ; ns | **115[66-236]**  AUC=0.46 ; ns | **181[151-196]**  AUC= 0.61 ; ns | **87[62-137]**  AUC=0.69 ; p<.05 | **152[98-236]** |
| **Device landing zone calcific weighted volume - RCC** | **155[87-237]**  AUC=0.59 ; ns | **110[55-248]**  AUC=0.48 ; ns | **214[131-260]**  AUC= 0.74 ; p<.01 | **85[51-139]**  AUC=0.65 ; ns | **117[67-197]** |
| **Device landing zone calcific weighted volume – LCC** | **126[65-235]**  AUC=0.54 ; ns | **102[80-193]**  AUC=0.48 ; ns | **126[82-242]**  AUC= 0.54 ; ns | **92[49-151]**  AUC=0.59 ; ns | **135[72-199]** |
| **Leaflet Calcification Volume (mm^3^)** | **376[223-784]**  AUC=0.52 ; ns | **475[226-920]**  AUC=0.49; ns | **718[468-995]**  AUC=0.69 ; ns | **350[199-481]**  AUC=0.63 ; ns | **519[307-760]** |
| **Annular/LVOT Calcification Volume**  **(mm^3^)** | **37[3.6-147]**  AUC=0.66 ; ns | **15[2.9-115]**  AUC=0.58; ns | **14[0.5-74]**  AUC=0.50 ; ns | **0[0-20]**  AUC=0.65 ; ns | **5[0-55]** |
| **Calcification Grade**  **(none-severe)** | **None: 1 (0.8%)**  **Mild: 7 (5.3%)**  **Moderate: 8 (6%)**  **Severe: 10 (7.5%)**  AUC=0.68 ; ns | **None: 7 (5.3%)**  **Mild: 1 (0.8%)**  **Moderate: 1 (0.8%)**  **Severe: 2 (1.5%)**  AUC=0.54 ; ns | **None: 5 (3.8%)**  **Mild: 3 (2.3%)**  **Moderate: 1 (0.8%)**  **Severe: 1 (0.8%)**  AUC=0.58 ; ns | **None: 17 (12.8%)**  **Mild: 2 (1.5%)**  **Moderate: 0 (0%)**  **Severe: 0 (0%)**  AUC=0.61 ; ns | **None: 39 (29.3%)**  **Mild: 12 (9%)**  **Moderate: 13 (9.8%)**  **Severe: 3 (2.3%)** |
| **Agatston Score**  **(AU)** | **2973[2449-4609]**  AUC=0.54 ; ns | **2384[1926-3816]**  AUC=0.59 ; ns | **4601[3640-5524]**  AUC= 0.83 ; p<.01 | **2476[2154-3109]**  AUC=0.41 ; ns | **2726[2141-3682]** |
| **Valve Size**  **(20-29 mm)** | **20mm: 1 (0.8%)**  **23mm: 3 (2.3%)**  **26mm: 11 (8.3%)**  **29mm: 11 (8.3%)**  AUC=0.63 ; ns | **20mm: 1 (0.8%)**  **23mm: 5 (3.8%)**  **26mm: 3 (2.3%)**  **29mm: 2 (1.5%)**  AUC=0.64 ; ns | **20mm: 0 (0%)**  **23mm: 3 (2.3%)**  **26mm: 3 (2.3%)**  **29mm: 4 (3%)**  AUC=0.55 ; ns | **20mm: 2 (1.5%)**  **23mm: 6 (4.5%)**  **26mm: 8 (6%)**  **29mm: 3 (2.3%)**  AUC=0.61 ; ns | **20mm: 1 (0.8%)**  **23mm: 14 (10.5%)**  **26mm: 38 (28.6%)**  **29mm: 14 (10.5%)** |
| **Area Cover Index (%)** | **12.5[7.7-17.9]**  AUC=0.73 ; p<.01  OR(95%CI)=1.11(1.04-1.19) | **9.5[2.6-13.4]**  AUC=0.62; ns | **3.24[-2.4-8.8]**  AUC= 0.63 ; ns | **1.83[-3.3-9.3]**  AUC=0.65 ; ns | **5[1.2-11]** |

| Multivariate Linear Regression_N=33_ | Event/Outcome | Model 1 | | | Model 2 | | Model 3 | | | |
| --- | --- | --- | --- | --- | --- | --- | --- | --- | --- | --- |
|  |  | DLZ intensity weighted calcification volume | | | Calcification volume | | Conventional clinical parameters | | | |
| Statistical Tests |  | NCC | RCC | LCC | Leaflet | ALVOT | Calcification grade | Agatston score | Valve Size | Area Cover Index |
| R^2^ | DNA (%) | 0.561 (p<.05) | | | 0.528 (p<.01) | | 0.509 (p<.001) | | | |
| F-score/RMSE | DNA (%) | 3.26/6.9 | | | 4.85/7.15 | | 7.25/7.3 | | | |
| Parameter Comparison^*^  $(F, pval$) | Model 1 | 1.44  (ns) | 0.8  (ns) | 0.08  (ns) | 2.17  (ns) | 0.7  (ns) | 7.6  (p<.05) | 0.81  (ns) | 6.16  (p<.05) | 1.15  (ns) |
|  | Model 2 | -- | -- | -- | 1.0  (ns) | 0.00  (ns) | 8.26  (p<.01) | 0.43  (ns) | 11.02  (p<.01) | 2.82  (ns) |
|  | Model 3 | -- | -- | -- | -- | -- | 8.75  (p<.01) | 3.27  (ns) | 10.48  (p<.01) | 3.05  (ns) |
| Model Comparison^**^  $(\Delta R^{2}, pval$) | Model 1 vs Model 2 | 0.02 (ns) | | | | | | | | |
|  | Model 1 vs Model 3 | 0.05 (ns) | | | | | | | | |
|  | Model 2 vs Model 3 | 0.03 (ns) | | | | | | | | |
