# Supplementary figures and images for: Incremental prognostic value of intensity-weighted regional calcification scoring using contrast CT imaging in TAVR
Source: Eur Heart J Imaging Methods Pract. 2023 Sep 29;1(2):qyad027. doi: 10.1093/ehjimp/qyad027 (PMC11195730; doi:10.1093/ehjimp/qyad027)

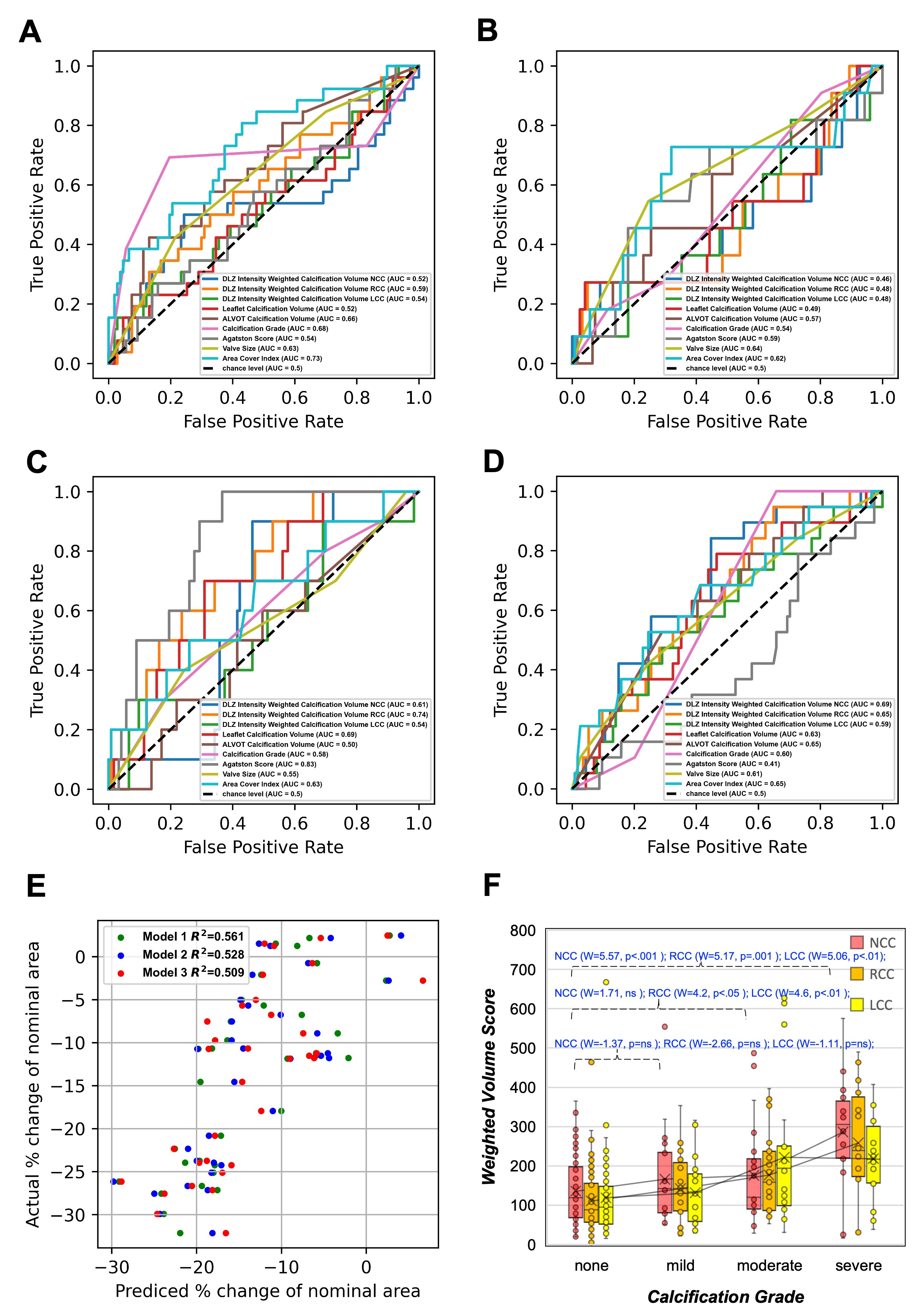

Supplement: qyad027_Supplementary_Data [file qyad027_Supplementary_Data.zip › Fig S1.jpg]

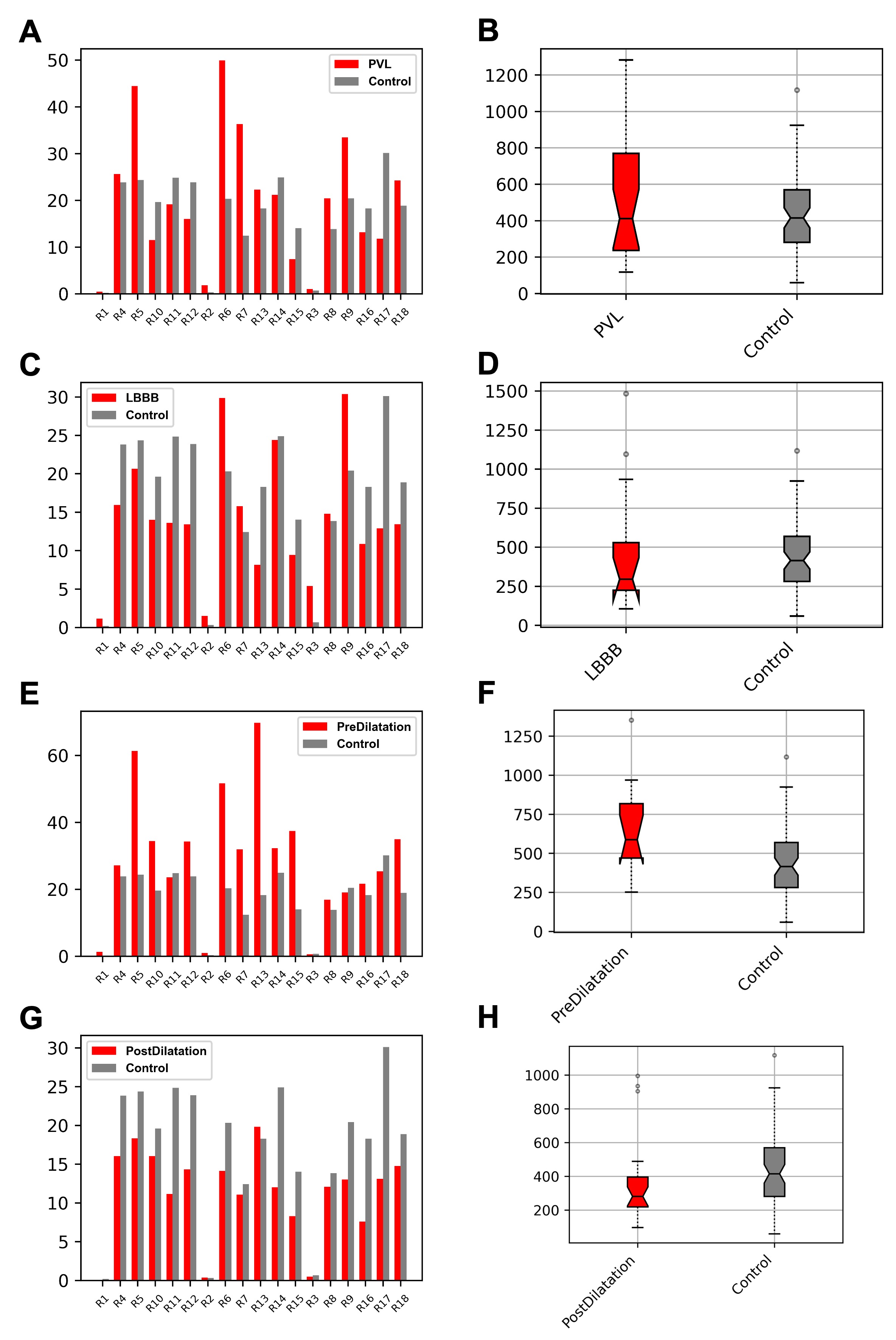

Supplement: qyad027_Supplementary_Data [file qyad027_Supplementary_Data.zip › Fig S2.jpg]

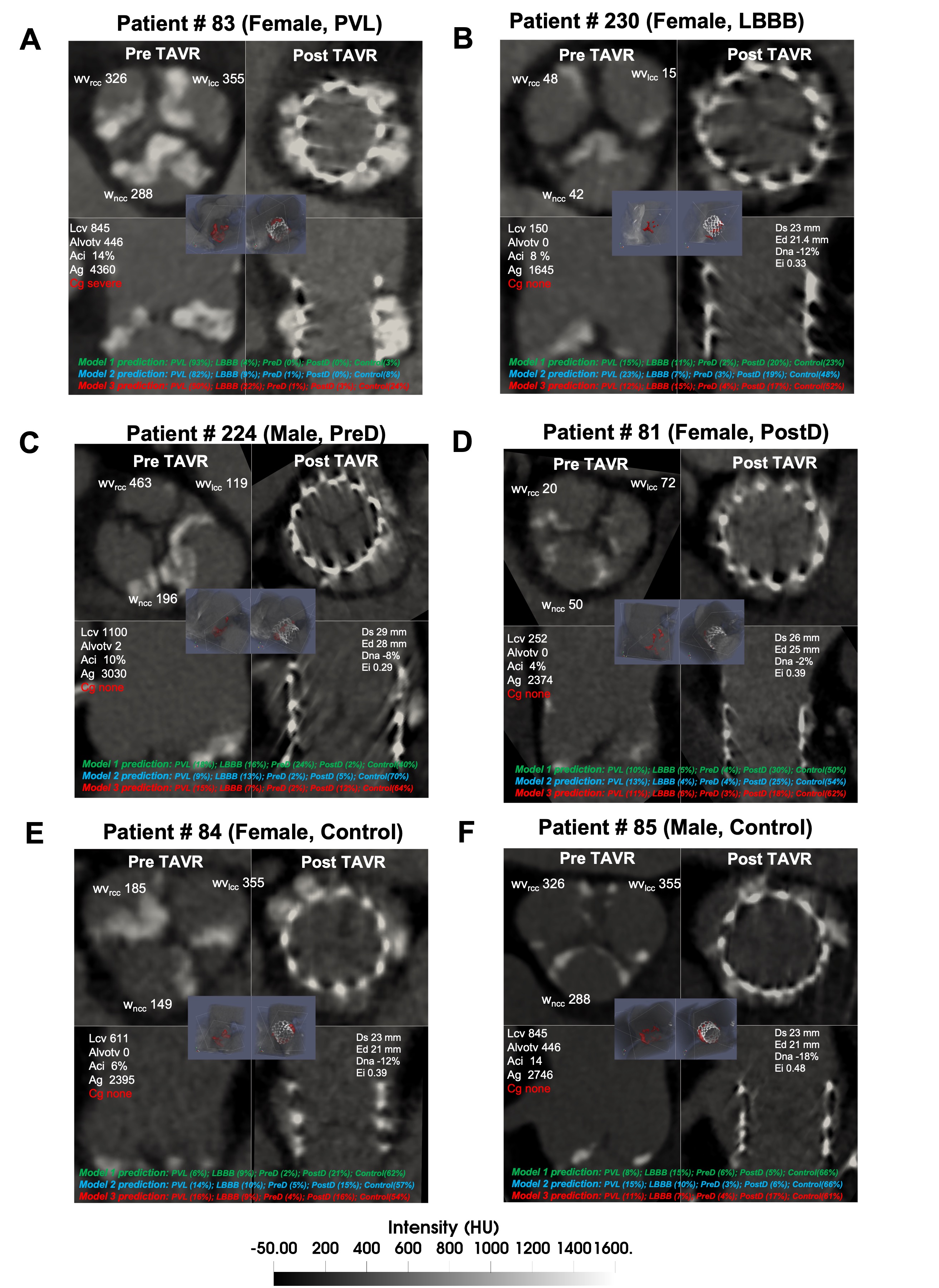

Supplement: qyad027_Supplementary_Data [file qyad027_Supplementary_Data.zip › Fig S3.jpg]
